# The long term outcome of micturition, defecation and sexual function after spinal surgery for cauda equina syndrome

**DOI:** 10.1371/journal.pone.0175987

**Published:** 2017-04-19

**Authors:** Nina S. Korse, Anna B. Veldman, Wilco C. Peul, Carmen L. A. Vleggeert-Lankamp

**Affiliations:** 1 Department of Neurosurgery, Leiden University Medical Center, Leiden, the Netherlands; 2 Department of Neurosurgery, Haaglanden Medical Center, the Hague, the Netherlands; George Washington University, UNITED STATES

## Abstract

**Background:**

Cauda equina syndrome (CES) is a rare neurologic complication of lumbar herniated disc for which emergency surgical decompression should be undertaken. Despite the common belief that the restoration of functions that are affected by CES can take several years postoperatively, follow up seldom exceeds the first year after surgery. Long term outcome of especially micturition, defecation and sexual function—which are by definition affected in CES—are unknown. The aim of this study is to evaluate 1) postoperative long term outcome of micturition, defecation and sexual function in CES patients 2) attitude of patients towards received hospital care with regard to (recovery of) these functions.

**Methods:**

CES patients were selected by screening the records of all patients operated on lumbar herniated disc in our university hospital between 1995–2010. A questionnaire was sent to the selected CES patients evaluating current complaints of micturition, defecation and sexual function and attitude towards delivered care with focus on micturition, defecation and sexual function.

**Results:**

Thirty-seven of 66 eligible CES patients were included (response rate 71%, inclusion rate 56%). Median time after surgery was 13.8 years (range 5.8–21.8 years). Dysfunction at follow up was highly prevalent: 38% micturition dysfunction, 43% defecation dysfunction and 54% sexual dysfunction. Younger age at presentation was associated with sexual dysfunction at follow up: for every year younger at presentation, odds ratio for sexual dysfunction at follow up was 1.11 (*p* = 0.035). Other associations with outcome were not identified. Two-third of the CES patients wished their neurosurgeon had given them more prognostic information about micturition, defecation and sexual function.

**Conclusion:**

The presented data demonstrate that dysfunction of micturition, defecation and sexual function are still highly prevalent in a large number of CES patients even years postoperatively. These alarming follow up data probably have a devastating effect on personal perceived quality of life, which should be studied in more detail. CES patients communicate a clear demand for more prognostic information. The presented figures enable clinicians to inform their CES patients more realistically about long term postoperative outcome of micturition, defecation and sexual function after surgical intervention.

## Introduction

Cauda equina syndrome (CES) is a rare condition caused by compression of several nerve roots of the cauda equina, including lower sacral nerves influencing the bladder, rectal and genital function, most often due to a herniated disc.[1] It’s classical presentation consists of loss of sensation of the saddle area, sphincter dysfunction (bladder and/or bowel) and/or sexual dysfunction, often in combination with motor deficit with or without reflex changes of the lower limbs.[1,2] Diagnosis is mostly performed on clinical grounds followed by magnetic resonance (MR) or computed tomography (CT) imaging demonstrating the exact location of compression and its causal element. Surgical spinal decompression by bone and herniated disc removal is the only effective measurement that should be taken as soon as possible in case CES is diagnosed. The influence of timing of surgery on outcome has been a topic of hot debate in literature and there is now substantial evidence that decompression within 48 hours yields significantly better outcomes than decompression after 48 hours.[3]

Literature about evidence in timing of surgery for CES is limited in two distinctive ways. Firstly, outcome measurements in published studies are mainly concentrated around bladder function, motor function, general quality of life and sciatic pain. Details about outcome of defecation and/or sexual function are only marginally described, even though those functions are, by definition, often impaired in patients with CES.[2,4–7] Reasons for this trend are varying, but especially the embarrassment accompanying the conversation about these topics–from the perspective of the patient as well as the doctor–should not be underestimated.

Secondly, follow-up of CES patients by neurosurgeons or neurologists rarely passes the first few years after first encounter. Obviously, some studies do include individual patients with follow up of several years, however, those patient numbers are small (*n*≤8 patients)[8,9] and evaluation of outcome seldom includes defecation and sexual function.[10–23] Since it is known that both urethral and anal sphincter function can improve even after several years post-surgery,[8,10,24,25] it seems sensible to evaluate long term outcome up to a decade or more after decompressive surgery, next to the evaluation after one or two years.

The lack of information about long term recovery of micturition, defecation and sexual function is bothersome in the case of CES in particular. CES patients pre-eminently face a long period of recovery, and the lack of prognostic data prevents the clinician from informing CES patients about recovery prospects of micturition, defecation and sexual dysfunction. This poses the patient in a position of maximum uncertainty. The fact that micturition, defecation and especially sexual function are topics which are difficult to discuss, makes the patient and his or her partner even more prone to discomfort and isolation when experiencing complaints.

Aims of this study are to evaluate 1) the outcome of CES after decompressive surgery with a minimum follow up of several years, and with a particular interest in micturition, defecation and sexual function; 2) predictors for outcome of these functions; 3) the attitude of CES patients toward delivered hospital care before and after decompressive surgery.

## Material and methods

In a previous publication[7] the authors described a cohort of 75 patients with cauda equina syndrome (CES), focusing on presentation and outcome up to several months postoperatively. The cohort was selected through screening medical records of patients who had had surgery in the Leiden University Medical Center (LUMC; referral hospital for spinal surgery) with surgery code “lumbar discectomy” or “recurrent lumbar discectomy” between January 1995 and September 2010. According to consensus of literature, definition of CES was set by presence of one or more of the following: 1) dysfunction of micturition and/or defecation, 2) altered sensation of the saddle area, 3) sexual dysfunction, with possible neurologic deficit in the lower limbs (motor or sensory loss or reflex changes).[1,2]

For the current study, contact details of all 75 patients were traced after approval of the local medical ethical committee was granted. In case of death, elusive contact details or otherwise inability to communicate, a patient was considered ineligible for the study. Patients were sent a questionnaire (hard copy) with an accompanying letter explaining the contents of the study and an informed consent form that had to be returned together with the questionnaire. The questionnaire (not validated) covered the following items: 1) medical history; 2) whether complaints of micturition, defecation, sexual dysfunction, altered sensation of the saddle area and/or sciatica were discussed at first presentation to the neurosurgeon; 3) whether complaints of micturition-, defecation- and sexual dysfunction were present at the time of the postoperative visit at the outpatient department (by default 6 weeks after surgery); 4) whether the neurosurgeon had paid enough attention to aforementioned complaints during the visit at the outpatient department; 5) whether complaints of micturition, defecation and/or sexual dysfunction are currently present; 6) whether the neurosurgeon–before or after decompressive surgery–had said anything about the prognosis of micturition, defecation and/or sexual function; 7) whether the patient had wished for more information from the neurosurgeon about the prognosis of micturition, defecation and sexual function.

After the initial invitation by hard copy mail, patients which had not sent back the questionnaire were contacted by telephone and asked whether they wanted to participate in the study. If so, a second questionnaire was sent (hard copy). Data of Case Record Forms were collected in Excel and imported in IBM SPSS Statistics version 23.0.

In addition, the following patient characteristics were collected from the medical file: gender; age at surgery; level of herniated disc according to file; duration of complaints of herniated disc at presentation (defined by onset of sciatica); duration of CES complaints at presentation; micturition/defecation/sexual dysfunction at presentation according to file; time between presentation at first doctor and decompressive surgery (in hours). To correlate the patients experiences with the medical file, medical notes about micturition/defecation/sexual function at follow up at the outpatient clinic were collected from the medical file as well.

### Statistical analysis

IBM SPSS Statistics version 23.0 was used for analysis. Comparing independent groups with categorical variables was done with Chi Square test; Mann-Whitney U test was used in case of numerical variables. For comparisons between paired groups of categorical variables, McNemar’s test was done. Binary logistic regression models were used to evaluate predictors for micturition, defecation and sexual dysfunction at long term follow up, with inclusion of the following variables: gender; age; duration of complaints of herniated disc at presentation; duration of complaints of CES at presentation; time to decompression. Dysfunction of defecation at presentation (according to the file) was added to the models for micturition and sexual dysfunction at follow up; dysfunction of micturition at presentation (according to the file) was added to the models for defecation and sexual dysfunction at follow up. Before running the regression models, missing data was imputed using five imputation sets for the following variables: duration of complaints of herniated disc (*n* = 2 missing); duration of complaints of CES (*n* = 2 missing); defecation dysfunction at presentation (*n* = 7 missing). For the regression model, time to decompression was stratified into six groups: <13 hours; 13–24 hours; 25–36 hours; 37–48 hours; 49–72 hours; >72 hours.

## Results

### Baseline

**Fig 1:**
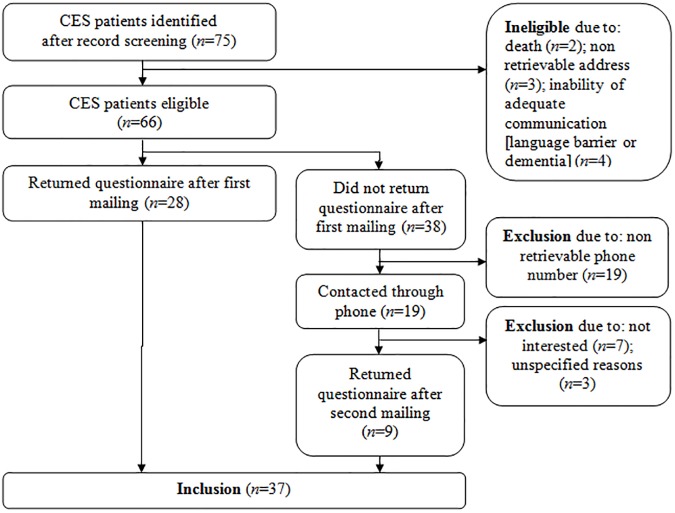
Inclusion of patients.

Thirty-seven patients were included (Fig 1). Response rate was 71% (10 additional patients responded with a wish to not participate). Patient characteristics (retrieved from the medical file) are depicted in Table 1.

**Table 1:** Patient characteristics at presentation (*n* = 37).

	*n*
**Male gender**	18 (48.6%)
**Mean age in years**	44.6 (SD 10.2; range 29–67)
**Median duration of complaints of herniated disc in days**^*I*^	30 (range 1–5110)
**Level of disc lesion according to file** ^*II*^	
L1-L2	1
L2-L3	2
L3-L4	1
L4-L5	12
L5-S1	21
L6-S1	1
**Median duration of complaints of CES in hours** ^*III*^	48
**Micturition dysfunction**	34 (91.9%)
**Altered sensation of the saddle area**	36 (97.3%)
**Sciatica**	35 (94.6%)
**Defecation dysfunction**^*III*^	23 (76.7%)
**Sexual dysfunction**^*IV*^	12 (100%)
**Decreased anal sphincter tone**^*V*^	18 (64.3%)
**Decreased anal sphincter reflex**^*VI*^	13 (56.5%)

^I^available for n = 35

^II^total adds up to 38 due to one patient with a double lesion (L2-L3 and L4-L5)

^III^available for n = 30

^IV^available for n = 12

^V^available for n = 28

^VI^available for n = 23.

Baseline characteristics of patients who responded (gender; age at surgery; duration of herniated disc complaints at presentation; duration of CES complaints at presentation; prevalence of dysfunction at presentation) were compared with non-responders and revealed no statistically significant differences (smallest *p*-value 0.174).

The majority of patients was referred to the LUMC by neurologists from referring hospitals (*n* = 29). The remaining were referred by neurologists from the LUMC (*n* = 6), by doctors in the accidents & emergency department of the LUMC (*n* = 1) or by the general practitioner (*n* = 1). All patients were surgically decompressed. Time to decompression was 7–12 hours (*n* = 2), 13–24 hours (*n* = 21), 25–48 hours (*n* = 11), 49–72 hours (*n* = 1) or >72 hours (*n* = 2). Surgical decompression of the latter two patients was delayed primarily by the first doctor where they presented: decompressive surgery took place within 24 hours after first presentation at the neurosurgeon (exact start of CES complaints before presentation was not retrievable from the medical file) and within 72 hours after first presentation at the neurosurgeon (start of CES complaints was 21 days before presentation: extreme patient delay).

When asked what they had discussed with the neurosurgeon at presentation, most patients mentioned altered sensation of the saddle area (*n* = 24), sciatica (*n* = 24) and dysfunction of micturition (*n* = 24). Only 9 patients reported to have discussed dysfunction of defecation. One patient did not answer this question with regard to sexual dysfunction, of the remaining 36 patients, 5 indicated to have discussed sexual dysfunction at the moment of presentation (of whom 2 male and 3 female).

### Follow up at the outpatient department

The median time between surgery and follow-up of CES patients at the outpatient department (FU OPD) was 56 days postoperatively. One respondent did not answer any question about FU OPD, two additional respondents did merely not answer the question about sexual dysfunction at FU OPD. Patient reported data (retrieved from the questionnaires) and doctor reported data (retrieved from the medical files) are mentioned separately and, in addition, are compared to each other.

#### Micturition

Patient reported data demonstrated 58.3% (21/36) dysfunction of micturition. Doctor reported data displayed dysfunction in 37.0% (27 files with micturition documentation, 10 marked as dysfunction). The differences in reporting dysfunction between patient and doctor data did not reach statistical significance(*p* = 0.289).

#### Defecation

Patient reported data displayed 47.2% (17/36) dysfunction of defecation. Doctor reported data demonstrated dysfunction in 23.8% (21 files with defecation documentation, 5 marked as dysfunction). The differences in reporting dysfunction between patient and doctor data did not reach statistical significance (*p* = 0.219).

#### Sexual dysfunction

Patient reported data revealed 55.9% (19/34) sexual dysfunction (of whom 9 male and 10 female). Doctor reported data were lacking, since only 7 files contained documentation about sexual function (5 male and 2 female): 4 were indicated to have dysfunction (3 male, 1 female). The differences in reporting dysfunction between patient and doctor data revealed no statistical significant differences (*p* = 1.000).

Whether the neurosurgeon had paid enough attention to complaints of micturition, defecation and sexual function at FU OPD was answered by 28/37 patients (no response *n* = 4, ‘not applicable’ *n* = 5). One quarter (7/28) judged that the neurosurgeon did not pay enough attention to their complaints at FU OPD; 5 of them were female.

### Long term follow up

The median follow up time at the moment of answering the questionnaire was 13.8 years after decompressive surgery (range 5.8–21.8 years). Mean age at long term follow up was 57.8 years (SD 11.6). None of the patients reported current medical conditions likely to influence micturition and defecation. Three patients reported to suffer from diabetes mellitus, which was considered by the authors as a disease possibly influencing sexual function.

#### Micturition

Micturition dysfunction secondary to CES was present in 37.8% (14/37). Complaints that were mentioned: catheterization (*n* = 3); incontinence (*n* = 1); abnormal sensation of voiding (*n* = 3); combination of the latter two (*n* = 4); inability to void completely (*n* = 1); combination of abnormal sensation of voiding, incontinence and unable to void completely (*n* = 1); not specified (*n* = 1). Another 5 patients reported ‘new’ complaints that were not present at FU OPD and thus not designated as caused by CES (designated causes: prostate problems [*n* = 3], gynaecological prolapse [*n* = 1], surgery [*n* = 1]). One additional patient (male, 52 years old) indicated micturition problems at FU OPD but mentioned dripping as his only current complaint, which was regarded as prostate problems.

#### Defecation

Defecation dysfunction secondary to CES was present in 43.2% (16/37). Complaints that were mentioned: abnormal sensation of passing stool (*n* = 4); abnormal sensation of passing stool and incontinence (*n* = 1); manual evacuation of stool (*n* = 2); constipation (*n* = 1); combination of constipation with abnormal sensation of passing stool (*n* = 2), incontinence (*n* = 1) or uncontrolled flatus (*n* = 1); not specified (*n* = 4).

#### Sexual dysfunction

Sexual dysfunction believed to be secondary to CES was present in 54.3% (19/35), of whom 9 male and 10 female (NB: one of those patients indicated to suffer from diabetes mellitus). Complaints were: dysaesthesia of the genital region (*n* = 8); combination of dysaesthesia of the genital region with problems to reach orgasm (*n* = 3) or with erectile dysfunction (*n* = 4); delayed erection and orgasm (*n* = 1); not specified (*n* = 3).

Prevalences of dysfunction were compared between short (FU OPD) and long term follow up; for micturition dysfunction, it had decreased significantly (Table 2; *p* = 0.008).

**Table 2:** Proportion of patients with complaints: comparison between short and long term follow up.

	At FU OPD	At long term follow up	*p*-value
Micturition dysfunction	58.3% (21/36)	36.1% (13/36)	0.008
Defecation dysfunction	47.2% (17/36)	41.7% (15/36)	0.500
Sexual dysfunction	55.9 (19/34)	52.9 (18/34)	1.000

NB: due to some missing responses for FU OPD, for these comparisons n = 36 and n = 34 instead of n = 37

### Information about prognosis

**Fig 2:**
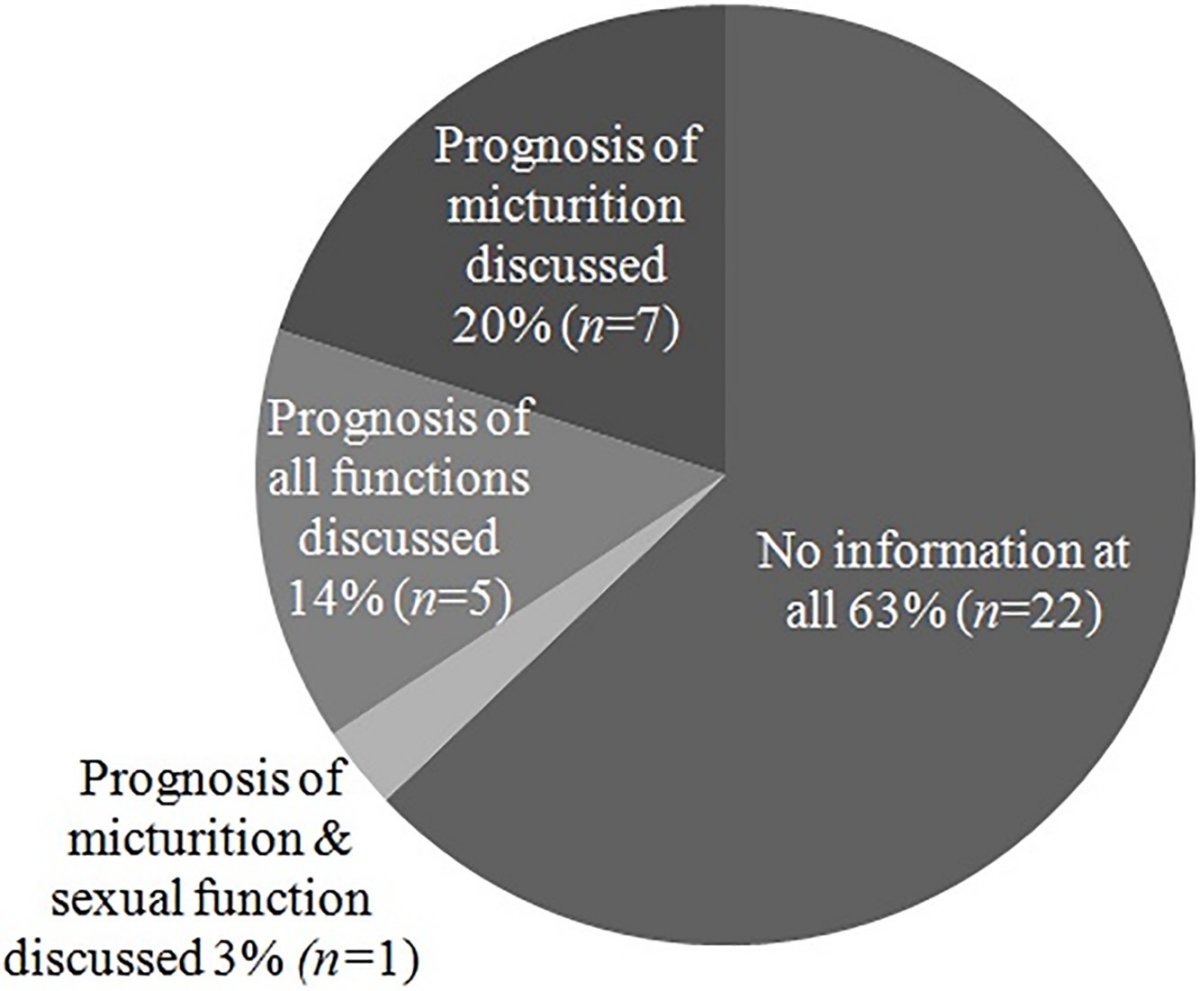
Did you receive information about prognosis of recovery of function(s)?

Two-third (22/35) of patients indicated not to have received any information from the neurosurgeon–before or after surgery–about the recovery of micturition, defecation and/or sexual function (Fig 2). More and/or better information from the neurosurgeon about the recovery of functions was demanded by 23 patients (65.7%).

### Predictors for long term outcome

Due to quasi-complete separation of the data of micturition dysfunction at presentation (sign was present in almost all patients), this variable could not be included in the regression models as a potential predictor. None of the tested variables (gender; age; duration of complaints of herniated disc at presentation; duration of complaints of CES at presentation; time to surgery) were identified as a predictor for long term outcome of micturition or defecation. Younger age at presentation was significantly associated with more sexual function dysfunction at follow up: for every year younger at presentation, odds ratio for sexual dysfunction at long term follow up was 1.11 (*p* = 0.035).

Next to the outlined regression analysis, the cohort was evaluated in detail with regard to two presenting characteristics that were described by others to be of predictive value for worse outcome: 1) complete saddle anesthesia and[17,19] and 2) significant sphincter dysfunction (defined as either necessary urinary catheterization at presentation for bladder dysfunction and as decreased anal sphincter tension in combination with absent anal sphincter reflex for bowel dysfunction)[17]. With regard to the first characteristic: six of our patients presented with complete saddle anesthesia. At long term follow up, all 6 patients (100%) reported defecation dysfunction and 4 of them (66.7%) reported micturition dysfunction. Of the 31 patients without complete saddle anesthesia at presentation, 10 (32.3%) reported micturition dysfunction at follow up and 10 (32.3%) reported defecation dysfunction at follow up.

NB: the total number of patients with micturition or defecation dysfunction was 19, including 11 suffering from both.

Secondly, significant sphincter dysfunction at presentation, thus either 1) necessary bladder catheterization or 2) decreased anal sphincter tone in combination with absent anal sphincter reflex, was evaluated in our cohort. Fifteen patients were given a urinary catheter at presentation; 5 of them (33.3%) reported micturition dysfunction at follow up. Of the 22 patients not being given a urinary catheter at presentation, 9 (40.9%) reported micturition dysfunction at final follow up. Worth mentioning, with regard to evaluating the predicting value of urinary catheterization at presentation, is that reasons for catheterization are varying among patients: e.g., it does not substantiate the amount of dysfunction but might be used as a preventive or diagnostic tool as well. Anal sphincter tone and reflex were not documented for every patient (Table 1); of the patients with documentation, 11 patients were reported to have both reduced tone and absent reflex. Of those 11 patients, 6 (54.5%) reported defecation dysfunction at long term follow up. As a control group, the patients with at least one of the two (either anal sphincter tone or reflex) to be documented as normal at presentation, were evaluated; this were 16 patients. Four (25%) reported defecation dysfunction at follow up. The remaining 10 patients that were not evaluated in this respect were patients without documentation of both anal sphincter tone and reflex at presentation or with one of the two being positive and the other one not documented.

## Discussion

Although CES patients undergo acute surgical decompression as a salvage procedure for their deteriorating or absent urinary, defecation and genital function, outcome is usually not evaluated in follow up visits. The presented results of this retrospective survey are alarming as at least one third of patients report micturition problems and about half of all patients complain about defecation- and sexual dysfunction years after surgery.

Micturition dysfunction decreases significantly between the follow up at the outpatient clinic (median 56 days post-surgery) and at long term follow up (median 13.8 years post-surgery), from 58.3% to 36.1% (*p* = 0.008).

### Findings in relation to literature

Earlier studies suggested that recovery of genito-urinary and rectal functions is possible even several years after decompressive surgery.[8,10,24,25] Up to date, only a few studies have evaluated both micturition, defecation and sexual function after decompressive surgery for CES. The reliability of those results are restricted by small patient cohorts[26] and extremely delayed decompression[27]. The study of McCarthy et al evaluated outcome of both micturition, defecation and sexual function in a cohort of 42 CES patients with a shorter follow up time than the current study (mean 5 years, minimum 2.1 years), demonstrating similar rates of dysfunction of micturition (36%), but slightly higher rates of dysfunction of defecation (60%) and sexual function (57%).[28] The higher rates of defecation and sexual dysfunction in the cohort of McCarthy et al compared to that of the presented cohort, might suggest that improvement is still possible several years post-surgery.

Also in the current study, it is displayed that (patient-reported) dysfunction of all three functions is higher at FU OPD than at long term follow up. Since all figures are reported by patients, the possibility of tolerance of complaints over time and therefore, a reported lower rate of dysfunction, should be taken into account.

In our study, younger age at presentation was associated with sexual dysfunction at long term follow up (OR 1.11 for every year younger at presentation; *p* = 0.035). This was not described earlier. This finding is most likely due to the higher frequency of sexual activity of younger patients, making them more prone to notice and report sexual dysfunction; indeed, decreasing sexual desire in elderly women was reported by Hayes earlier.[29] In our patients, frequency of sexual activity was not evaluated in a structured manner.

Time to decompression is the best described predictor in CES.[3,17,18,30–33] In the presented cohort, time to decompression was included in the regression analysis as a possible confounder, yet was not found to be significantly associated with outcome of evaluated function. There are multiple reasons for this, such as 1) relatively small patient cohort (several studies reporting an association were meta-analyses[3,31,33]); 2) outcome was separately evaluated for micturition, defecation and sexual function instead of evaluated in a combined matter; 3) relatively few patients were decompressed beyond 48 hours, which was the break point in several studies[3,30,33]. Interestingly, none of the included patients were decompressed within 6 hours. Reason might be logistics: patients’ first presentation was often in a referral hospital.

Literature describes several other predictors. Kennedy et al evaluated 19 CES patients with a minimum follow up of 1.8 years after decompressive surgery, identifying five patients with poor outcome, with poor outcome defined as any residual deficit regarded as physical or psychological impairment.[17] One of the predictors identified was delayed decompression (>24 hours). Another predictor found was complete perianal anesthesia at presentation: seven out of 19 patients suffered from this, including all five with poor outcome. Third predictor was significant sphincter dysfunction at presentation (bladder or bowel). Significant was defined as urinary catheterization in case of bladder dysfunction (12/19) and as decreased anal sphincter tension and absent anal sphincter reflex in case of bowel dysfunction (15/19): of the five patients with poor outcome, five demonstrated significant bladder sphincter dysfunction and four demonstrated significant bowel sphincter dysfunction at presentation.

We evaluated our cohort in detail with regard to the latter two predictors that were identified by Kennedy et al; this was outlined in the results section. Of important note is that in our study, no binary overall outcome measurement was used as Kennedy et al did (e.g. poor and satisfactory), which makes the results of Kennedy not directly translatable to our results. Since micturition and defecation dysfunction were separate outcome measurements in our study, it seemed sensible to evaluate the patients that were given a urinary catheter at presentation for micturition dysfunction at follow up and the patients with decreased anal sphincter tone in combination with absent anal sphincter reflex for defecation dysfunction at follow up.

Summarizing the evaluations of our cohort it can be concluded that: 1) patients presenting with complete saddle anesthesia do seem more at risk for micturition and defecation dysfunction at follow up (66.7% versus 32.3% and 100% versus 32.3%, respectively); 2) patients being catheterized at presentation do not seem to be more at risk for micturition dysfunction at follow up (33.3% versus 40.0%)–not unlikely due to the fact that catheterization at presentation is not a distinctive characteristic of dysfunction per se; 3) patients with reduced anal sphincter tone and absent anal sphincter reflex do seem more at risk for defecation dysfunction at follow up (54.5% versus 25%). However, no firm conclusions can be drawn from these figures since they were not analyzed through statistics. This was not done because it would create unreliable regression models: adding the parameters complete perianal anesthesia at presentation, catheterization at presentation and reduced anal sphincter tension plus absent reflex at presentation to our regression models for outcome of micturition and defecation dysfunction would lead to overfitting (i.e. when a model consists of more parameters than events). Univariate analysis would be inappropriate due to the high risk of confounding (which risk is significantly reduced by using (multivariate) regression models, as was done in this study). Only in a larger cohort of patients (with thus more events), more parameters can be reliably added to the regression model.

Buchner et al presented a cohort of 22 CES patients with a mean follow up of 3.8 years postoperatively and mentioned absence of complete perianal anesthesia at presentation and female gender both being predictors of a better postoperative outcome. Postoperative outcome was graded by level of micturition and divided into four groups: excellent, good, fair or poor.[19] In the study of Buchner et al, outcome was regarded as “better” when the outcome shifted in the direction of excellent, regardless where it came from and how much it shifted in that direction. Doubtless, this is a completely different outcome measurement than the one that was used in the current cohort, and therefore not unlikely, inducing different correlations. In addition, the analysis by Buchner et al was univariate, which is more prone to confounding than the use of multivariate analysis as was done in the current study.

McCarthy et al evaluated 42 CES patients with a mean of 5 years after decompressive surgery and demonstrated 1) female gender to be a predictor of urinary incontinence at follow up and 2) bowel dysfunction at presentation to be a predictor of sexual dysfunction at follow up.[28] With regard to the first finding: in our regression analysis, no differentiation was made between urinary incontinence and other micturition problems at follow up. Evaluating the outcome for micturition in our study closer, displays that the 6 patients with urinary incontinence at long term follow up, were all female; of the remaining 8 patients with micturition problems but without incontinence, 3 were female and 5 were male. Thus, indeed, female gender seems to be associated with urinary incontinence at follow up in our cohort as well, however, no association was seen for the total group with micturition dysfunction. McCarthy et al identified bowel dysfunction at presentation as a predictor of sexual dysfunction at follow up, which was not demonstrated in our study. McCarthy et al used univariate models and used Bonferroni correction for proper interpretation of *p*-values. The use of univariate models instead of multivariate models could however be an explanation of identifying a predictor which was not found in our study. We think that the multivariate regression analysis used in our study diminished the risk of confounding.

### Differences in reporting between doctor and patient

Micturition and defecation dysfunction at FU OPD were more often reported by the patient than by the doctor (did not reach statistical significance, probably due to small patient numbers). For sexual dysfunction, patient and doctor reported data are much more similar. Reason for this might be that doctors find it easier to discuss micturition and defecation and thus also get to know (and document) about non-symptomatic patients. Sexual dysfunction, however, is more difficult to discuss and therefore, is not often discussed when the patient doesn’t bring up the subject him- or herself. The obvious reason for a patient to bring up this topic is because complaints are present. Consequently, the doctors notes about sexual function are relatively more often about dysfunction than the notes about micturition and defecation, suggesting that discussing sexual dysfunction is a barrier, not only for the patient but for the doctor as well.[6]

### Implications

Apart from younger age which was associated with sexual dysfunction at follow up, no predictors were identified. This urges the clinician to be attentive to dysfunction during presentation and follow up in all CES patients, bearing in mind the presented results of alarming high rates of dysfunction still years after surgery.

The presented CES patients indicated to be in dire need of recovery prospects. The fact that the majority did not receive any information of this kind, marks the lack of prognostic data in CES literature. With the presented data as best available evidence, it is now possible to start informing CES patients properly.

Despite recommendations from the Consortium of Spinal Cord Medicine (2010)[34] to identify threats to sexual wellbeing in high risk spinal patients, discussing sexual dysfunction in CES patients did not get foothold in current practice yet, which is highly regrettable considering the presented prevalence of dysfunction. The authors advocate to identify sexual dysfunction in CES patients at an early stage. Bringing up the subject cannot be left to the patient and is the solemn responsibility of the doctor: too often, the patient is unaware of the link of CES with sexual dysfunction and is too ashamed to ask.

### Limitations

This study might seem the largest cohort of CES patients with long term results which was presented up to now, however the total number of patients is still relatively small compared to evaluation studies of other neurological diseases. This restricted cohort size prevents a good intervention-prognostic variable analysis to predict outcome.

Like all surveys, this questionnaire study faced the problem of non-responding. This study achieved a response rate of 71% with inclusion rate of 56%. To accommodate for the best response rate possible, postal surveys were sent instead of web-based surveys[35] and telephone reminders were used as a proven method to improve response rate.[36,37] The average response rate for patient surveys is about 60%, which figure is deducted from studies published in 1991,[36] whereas it is well-known that the response rates have been decreasing ever since, especially for surveys sent by healthcare professionals.[38] More importantly, surveys about sensitive subjects such as sexual dysfunction are proven to be prone to lower response rates and display a decreasing response rate over the years as well.[39] Worth mentioning, the current study has an extremely long follow up time up to more than 21 years, which makes it more likely that patients are less prone to participate. Baseline characteristics of responders and non responders were not significantly different, making response bias unlikely.[40]

Of course, long term follow up creates risks for recall bias; i.e., patients report events differently from the true course of events due to loss of memory on the concerning item. This is something that cannot be corrected for in the current study design; any evaluation of long term outcome will introduce a risk of recall bias.

Obviously, because of the considerable long follow up period, the mean age of included patients has increased substantially during follow up (from 44.6 to 57.8 years). Increasing age changes the prevalence of problems of micturition, defecation and sexual function in the general population, thus might have also influenced the prevalence of dysfunction in the study population. Effort was taken to correct where possible: current medication use and co-morbidity was taken into account wherever dysfunction was reported, and where necessary, correction was used: correction was used for reported complaints that were thought not to be caused by CES but by other diseases such as urological prostate or gynaecological prolapse problems. The authors believe that the risk of bias was therefore minimized in this respect.

## Conclusion

This study presents data about long term outcome of micturition, defecation and sexual function in CES after decompression and is unique in three aspects 1) markedly large cohort 2) lengthy follow up 3) integral evaluation of defecation and sexual function next to micturition. This report demonstrates dysfunction to be extremely common years after surgery and communicates a clear demand from CES patients for more information about their prognosis on those functions. Without doubt, the presented data adds substantially to the current knowledge about CES. It gives the clinician in spinal care the opportunity to inform CES patients realistically about long term recovery of micturition, defecation and sexual function after decompressive surgery. With regard to the seriousness of genito-urinary and defecation dysfunction and impact on quality of life, a prospective study is necessary to evaluate the risk of permanent deficit and to identify predictive variables, which can be influenced by intervention and personal guidance in rehabilitation.

## Supporting information

S1 File Questionnaire (Dutch). (PDF)

S2 File Translated questionnaire (in English). (PDF)

## References

[1] Fraser S, Roberts L, Murphy E. Cauda equina syndrome: a literature review of its definition and clinical presentation. Arch Phys Med Rehabil 2009;90 (11): 1964–8. doi: 10.1016/j.apmr.2009.03.021 19887225 10.1016/j.apmr.2009.03.021

[2] Korse NS, Jacobs WC, Elzevier HW, Vleggeert-Lankamp CL. Complaints of micturition, defecation and sexual function in cauda equina syndrome due to lumbar disk herniation: a systematic review. Eur Spine J 2013;22(5):1019–29. doi: 10.1007/s00586-012-2601-8 23238848 10.1007/s00586-012-2601-8 PMC3657037

[3] Ahn UM, Ahn NU, Buchowski JM, Garret ES, Sieber AN, Kostuik JP. Cauda equina syndrome secondary to lumbar disc herniation: a meta-analysis of surgical outcomes. Spine 2000;25:1515–22. 10851100 10.1097/00007632-200006150-00010

[4] Bin MA, Hong WU, Lian-shun JIA, Wen YUAN, Guo-dong SHI, Jian-gang SHI. Cauda equina syndrome: a review of clinical progress. Chin Med J 2009;122(10):1214–22. 19493474

[5] Gardner A, Gardner E, Morley T. Cauda equina syndrome: a review of the current clinical and medico-legal position. Eur Spine J 2011;20:690–7. doi: 10.1007/s00586-010-1668-3 21193933 10.1007/s00586-010-1668-3 PMC3082683

[6] Korse NS, Nicolai MPJ, Both S, Vleggeert-Lankamp CLA, Elzevier HW. Discussing sexual health in spinal care. Eur Spine J 2016;25:766–73. doi: 10.1007/s00586-015-3991-1 25962812 10.1007/s00586-015-3991-1

[7] Korse NS, Pijpers JA, van Zwet E, Elzevier HW, Vleggeert-Lankamp CLA. Cauda equina syndrome: presentation, outcome and predictors with focus on micturition, defecation and sexual function. Eur Spine J 2017;26;894–904. doi: 10.1007/s00586-017-4943-8 28102451 10.1007/s00586-017-4943-8

[8] Chang HS, Nakagawa H, Mizuno J. Lumbar herniated disc presenting with cauda equina syndrome. Long term follow-up of four cases. Surg Neurol 2000;53:100–5. 10713185 10.1016/s0090-3019(99)00180-9

[9] Raj D, Coleman N. Cauda equina syndrome secondary to lumbar disc herniation. Acta Orthop Belg 2008;74:522–7. 18811037

[10] Jennett WB. A study of 25 cases of compression of the cauda equina by prolapsed intervertebral discs. J Neurol Neurosurg Psychiat 1956;19:109–16. 13346384 10.1136/jnnp.19.2.109 PMC497193

[11] Shephard RH. Diagnosis and prognosis of cauda equina syndrome produced by protrusion of lumbar disk. Br Med J 1959;2(5164):1434–9. 14445833 10.1136/bmj.2.5164.1434 PMC1991054

[12] Scott PJ. Bladder paralysis in cauda equina lesions from disc prolapse. J Bone Joint Surg Br 1965;47:224–35. 14302723

[13] Spannare BJ. Prolapsed lumbar intervertebral disc with partial or total occlusion of the spinal canal. Acta Neurochirur (Wien) 1978;42(3–4):189–98. 10.1007/BF01405333 717070

[14] Tay ECK, Chacha PB. Midline prolapsed of a lumbar intervertebral disc with compression of the cauda equina. J Bone Joint Surg Br 1979;61(1):43–6. 154521 10.1302/0301-620X.61B1.154521

[15] Kostuik JP, Harrington I, Alexander D, Rand W, Evans D. Cauda equina syndrome and lumbar disc herniation. J Bone Joint Surg Am 1986;68:386–91. 2936744

[16] Gleave JR, Macfarlane R. Prognosis for recovery of bladder function following lumbar central disc prolapse. Br J Neurosurg 1990;4(3):205–9. 2397046 10.3109/02688699008992725

[17] Kennedy JG, Soffe KE, McGrath A, Stephens MM, Walsh MG, McManus F. Predictors of outcome in cauda equina syndrome. Eur Spine J 1999;8:317–22. doi: 10.1007/s005860050180 10483835 10.1007/s005860050180 PMC3611188

[18] Busse JW, Bhandari M, Schnittker JB, Reddy K, Dunlop B. Delayed presentation of cauda equina syndrome secondary to lumbar disc herniation: functional outcomes and health-related quality of life. CJEM 2001;3(4):285–91. 17610771 10.1017/s1481803500005789

[19] Buchner M, Schiltenwolf M. Cauda equina syndrome caused by intervertebral lumbar disc prolapse–mid term results of 22 patients and review of the literature. Neuro-Orthopedics 2000;27:55–64. 10.3928/0147-7447-20020701-12 12138958

[20] Hussain SA, Gullain RW, Chitnavis BP. Cauda equina syndrome: outcome and implications for management. Br J Neurosurg 2003;17(2):164–7. 12820760 10.1080/0268869031000109098

[21] Qureshi A, Sell P. Cauda equina syndrome treated by surgical decompression: the influence of timing on surgical outcome. Eur Spine J 2007;16:2143–51. doi: 10.1007/s00586-007-0491-y 17828560 10.1007/s00586-007-0491-y PMC2140120

[22] Domen PM, Hofman PA, van Santbrink H, Weber WEJ. Predictive value of clinical characteristics in patients with suspected cauda equina syndrome. Eur J Neurol 2009;16(3):416–9. doi: 10.1111/j.1468-1331.2008.02510.x 19490073 10.1111/j.1468-1331.2008.02510.x

[23] Todd NV. Causes and outcomes of cauda equina syndrome in medico-legal practice: a single neurosurgical experience of 40 consecutive cases. Br J Neurosurg 2011;25(4):503–8. doi: 10.3109/02688697.2010.550344 21513452 10.3109/02688697.2010.550344

[24] Aho AJ, Auranen A, Pesonen K. Analysis of cauda equina symptoms in patients with lumbar disc prolapse. Preoperative and follow-up clinical and cystometric studies. Acta Chir Scand 1969;135(5):413–20. 5354193

[25] Hellstrom P, Kortelainen P, Kontturi M. Late urodynamic findings after surgery for cauda equina syndrome caused by a prolapsed lumbar intervertebral disk. J Urol 1986;135(2):308–12. 3944866 10.1016/s0022-5347(17)45621-7

[26] Tamburelli FC, Genitiempo M, Bochicchio L, Donisi L, Ratto C. Cauda equina syndrome: evaluation of the clinical outcome. Eur Rev Med Pharmacol Sci 2014;18:1098–1105. 24763893

[27] Dhatt S, Tahasildar N, Tripathy SK, Bahadur R, Dhillon M. Outcome of spinal decompression in cauda equina syndrome presenting late in developing countries: case series of 50 cases. Eur Spine J 2011;20:2235–39. doi: 10.1007/s00586-011-1840-4 21594752 10.1007/s00586-011-1840-4 PMC3229723

[28] McCarthy MJH, Aylott CEW, Grevitt MP, Hegarty J. Cauda equina syndrome. Factors affecting long-term functional and sphincteric outcome. Spine 2007;32(2):207–16. doi: 10.1097/01.brs.0000251750.20508.84 17224816 10.1097/01.brs.0000251750.20508.84

[29] Hayes RD, Dennerstein L, Bennett CM, Koochaki PE, Leiblum SR, Graziottin A. Relationship between hypoactive sexual desire disorder and aging. Fertil Steril 2007;87(1):107–12. doi: 10.1016/j.fertnstert.2006.05.071 17081522 10.1016/j.fertnstert.2006.05.071

[30] Beculic H, Skomorac R, Jusic A, Alic F, Imamovic M, Mekic-Abazovic A, et al Impact of timing on surgical outcome in patients with cauda equina syndrome caused by lumbar disc herniation. Med Glas (Zenica) 2016;13(2):136–41. 27452326 10.17392/861-16

[31] DeLong WB, Polissar N, Neradilek B. Timing of surgery in cauda equina syndrome with urinary retention: meta-analysis of observational studies. J Neurosurg Spine 2008;8(4):305–20. doi: 10.3171/SPI/2008/8/4/305 18377315 10.3171/SPI/2008/8/4/305

[32] Dinning TA, Schaeffer HR. Discogenic compression of the cauda equina: a surgical emergency. Aust N Z J Surg 1993;63(12):927–34. 8285904 10.1111/j.1445-2197.1993.tb01721.x

[33] Todd NV. Cauda equina syndrome: the timing of surgery probably does influence outcome. Br J Neurosurg 2005;19(4):301–6;discussion 307–8. doi: 10.1080/02688690500305324 16455534 10.1080/02688690500305324

[34] Consortium for Spinal Cord Medicine. Sexuality and reproductive health in adult with spinal cord injury: a clinical practice guideline for health-care professionals. J Spinal Cord Med 2010;33(3):281–336. 20737805 10.1080/10790268.2010.11689709 PMC2941243

[35] Leece P, Bhandari M, Sprague S, Swiontkowski MF, Schemitsch EH, Tornetta P, et al Internet versus mailed questionnaires: a randomized comparison (2). J Med Internet Res 2004;6(3):e30 Erratum in: J Med Internet Res. 2004;6(4):e38. Corrected and republished in: J Med Internet Res. 2004;6(4):e39. doi: 10.2196/jmir.6.3.e30 15471756 10.2196/jmir.6.3.e30 PMC1550617

[36] Asch DA, Jedrziewski MK, Christakis NA. Response rates to mail surveys published in medical journals. J Clin Epidemiol 1997;50(10):1129–36. 9368521 10.1016/s0895-4356(97)00126-1

[37] Van Geest J, Johnson TP, Welch VL. Methodologies for improving response rates in surveys of physicians: a systematic review. Eval Health Prof 2007;30(4):303–21. doi: 10.1177/0163278707307899 17986667 10.1177/0163278707307899

[38] Cook JV, Dickinson HO, Eccles MP. Response rates in postal surveys of healthcare professionals between 1996 and 2005: an observational study. BMC Health Serv Res 2009;9:160 doi: 10.1186/1472-6963-9-160 19751504 10.1186/1472-6963-9-160 PMC2758861

[39] Hayes RD, Bennett C, Dennerstein L, Gurrin L, Fairley C. Modeling response rates in surveys of female sexual difficulty and dysfunction. J Sex Med 2007;4:286–95. doi: 10.1111/j.1743-6109.2007.00433.x 17367424 10.1111/j.1743-6109.2007.00433.x

[40] Philips AW, Reddy S, Durning SJ. Improving response rates and evaluating nonresponse bias in surveys: AMEE guide no. 102. Med Teach 2016;38(3):217–28. doi: 10.3109/0142159X.2015.1105945 26648511 10.3109/0142159X.2015.1105945

