# Supplementary material for: The long term outcome of micturition, defecation and sexual function after spinal surgery for cauda equina syndrome
Source: PLoS One. 2017 Apr 19;12(4):e0175987. doi: 10.1371/journal.pone.0175987 (PMC5397048; doi:10.1371/journal.pone.0175987)
Supplement: S1 File — (PDF) [file pone.0175987.s001.pdf]

Vragenlijst

Deze vragenlijst gaat in op eventuele klachten die u heeft (of heeft gehad) ten gevolge van het cauda equina syndroom. Wij verzoeken u de vragenlijst zo volledig mogelijk in te vullen.

Om de invloed van andere ziekten uit te sluiten, is het belangrijk te weten of u andere medische aandoeningen heeft.

1. Lijdt u aan een (of meerdere) medische aandoening(en), cauda equina syndroom uitgezonderd?
   - o Nee
   - o Ja, namelijk:……………………………………………………………………………………
     ……………………………………………………………………………………………….…
     ……………………………………………………………………………………………..….
     …………………………………………………………………………………………….…..

2. Toen u zich bij de neurochirurg meldde voorafgaand aan uw operatie, over welke klachten, gerelateerd aan uw cauda equina syndroom, heeft u toen met de neurochirurg gesproken? (meerdere antwoorden mogelijk)
   - o Plasklachten (incontinentie of juist niet kunnen plassen, niet voelen passeren van urine, urinekatheter);
   - o Problemen met ontlasting (incontinentie voor ontlasting of juist verstopping, niet voelen passeren van ontlasting);
   - o Seksuele problemen (niet kunnen krijgen van erectie, niet/moeilijk kunnen krijgen van orgasme, verminderd gevoel tijdens de seks, of andere klachten die invloed hadden op de seks waarvan u denkt dat ze te wijten zijn aan cauda equina syndroom);
   - o Pijnklachten in de benen of billen;
   - o Veranderd/verminderd/verdwenen gevoel in ‘rijbroekgebied’ (billen, heupen, dijen);
   - o Ik heb over geen van bovenstaande klachten met de neurochirurg gesproken.

3. Toen u voor het eerst op de polikliniek Neurochirurgie kwam <u>na de operatie</u>, had u toen plasklachten die te wijten waren aan het cauda equina syndroom? (bijvoorbeeld: urine-incontinentie of juist niet kunnen plassen, niet voelen passeren van urine, urinekatheter)
   - o Ja
   - o Nee

4. Toen u voor het eerst op de polikliniek Neurochirurgie kwam <u>na de operatie</u>, had u toen problemen met ontlasting die te wijten waren aan het cauda equina syndroom? (bijvoorbeeld: incontinentie voor ontlasting of juist verstopping, niet voelen passeren van ontlasting)
   - o Ja
   - o Nee

5. Toen u voor het eerst op de polikliniek Neurochirurgie kwam <u>na de operatie</u>, had u toen problemen met seks die te wijten waren aan het cauda equina syndroom? (bijvoorbeeld: niet kunnen krijgen van erectie, niet/moeilijk kunnen krijgen van orgasme, verminderd gevoel tijdens de seks)
   - o Ja
   - o Nee

6. Vond u dat de neurochirurg tijdens de poliklinische controle <u>na de operatie</u> voldoende aandacht had voor plasklachten, problemen met ontlasting en/of seksuele problemen?
   - o Ja
   - o Nee, onvoldoende aandacht voor: ……………………………………………….
   - o Niet van toepassing (ik had geen klachten hiervan)

Onderstaande vragen gaan over klachten <u>op dit moment</u>.

7. Heeft u <u>op dit moment</u> plasklachten, waarvan u denkt dat ze te wijten zijn aan het cauda equina syndroom? (bijvoorbeeld: urine-incontinentie of juist niet kunnen plassen, niet voelen passeren van urine, urinekatheter)
   - o Ja, namelijk:……………………………………………………..
   - o Nee

8. Heeft u <u>op dit moment</u> problemen met de ontlasting, waarvan u denkt dat ze te wijten zijn aan het cauda equina syndroom? (bijvoorbeeld: incontinentie voor ontlasting of juist verstopping, niet voelen passeren van ontlasting)
   - o Ja, namelijk:……………………………………………………..
   - o Nee

9. Heeft u <u>op dit moment</u> problemen met seks, waarvan u denkt dat ze te wijten zijn aan het cauda equina syndroom? (bijvoorbeeld: niet kunnen krijgen van erectie, niet/moeilijk kunnen krijgen van orgasme, verminderd gevoel tijdens de seks)
   - o Ja, namelijk:……………………………………………………..
   - o Nee

10. Heeft u voor of na de operatie uitleg gekregen van de neurochirurg over het herstel van plasklachten, problemen met ontlasting en seksuele problemen? (meerdere antwoorden mogelijk)
    - o Uitleg over herstel van plasklachten;
    - o Uitleg over herstel van ontlastingsproblemen;
    - o Uitleg over herstel van seksuele problemen;
    - o Ik heb geen uitleg over bovenstaande gehad.

11. Had u het prettig gevonden meer uitleg te krijgen van de neurochirurg over herstel van plasklachten, problemen met de ontlasting en seksuele problemen ten gevolgen van het cauda equina syndroom?
    - o Ja
    - o Nee

12. Wilt u op de hoogte worden gehouden als resultaten van deze studie worden gepubliceerd?
    - o Ja, per email. Mijn mailadres: ………………….
    - o Ja, per post
    - o Nee

Als er iets onduidelijk is voor ons in deze ingevulde vragenlijst, zouden wij u graag bellen en om uitleg vragen. Als u daar geen bezwaar tegen heeft, kunt u dan hier het telefoonnummer melden waarop u te bereiken bent? ……………..
