# Supplementary material for: The long term outcome of micturition, defecation and sexual function after spinal surgery for cauda equina syndrome
Source: PLoS One. 2017 Apr 19;12(4):e0175987. doi: 10.1371/journal.pone.0175987 (PMC5397048; doi:10.1371/journal.pone.0175987)
Supplement: S2 File — (PDF) [file pone.0175987.s002.pdf]

Questionnaire *(translated from Dutch)*

This questionnaire focuses on potential complaints that you might have (or have had) due to cauda equina syndrome. We kindly ask you to answer the questions below in great detail.

To exclude the influence of other diseases, it is important to know if you have other medical conditions.

1. Do you suffer from a (or more) medical condition(s), cauda equina syndrome not included?
   - o No
   - o Yes, namely:…………………………………………………………………………… ……………………………………………………………………………………………… ……………………………………………………………………………………………… ………………………………………………………………………………………………..

2. When you presented at the neurosurgeon prior to your surgery, did you discuss with the neurosurgeon any of the complaints below, related to your cauda equina syndrome? (multiple answers possible)
   - o Urinary complaints (incontinence, inability to urinate, reduced or absent sensation of passing urine, urinary catheter);
   - o Bowel complaints (incontinence, constipation, reduced or absent sensation of passing stool);
   - o Sexual problems (unable to get an erection, difficulty or inability to reach orgasm, decreased sensation during sex, or other complaints that affected sex and which you think are directly related to cauda equina syndrome);
   - o Pain in the legs or buttocks;
   - o Changed/reduced/absent sensation in the ‘saddle’ area (buttocks, hips, thighs);
   - o I didn’t discuss any of the aforementioned complaints with the neurosurgeon.

3. At the time of your first visit to the Neurosurgery outpatient clinic <u>after surgery</u>, did you experience any urinary complaints which were due to cauda equina syndrome? (for example: urinary incontinence, inability to urinate, reduced or absent sensation of passing urine, urinary catheter)
   - o Yes
   - o No

4. At the time of your first visit to the Neurosurgery outpatient clinic <u>after surgery</u>, did you experience any bowel complaints which were due to cauda equina syndrome? (for example: incontinence, constipation, reduced or absent sensation of passing stool)
   - o Yes
   - o No

5. At the time of your first visit to the Neurosurgery outpatient clinic <u>after surgery</u>, did you experience any sexual problems which were due to cauda equina syndrome? (for example: unable to get an erection, difficulty or inability to reach orgasm, decreased sensation during sex, or other complaints that affected sex and which you think are directly related to cauda equina syndrome)
   - o Yes
   - o No

6. Do you think that the neurosurgeon during your visit at the outpatient clinic <u>after surgery</u> paid enough attention to urinary complaints, bowel complaints and/or sexual problems?
   - o Yes
   - o No, paid insufficient attention to: ……………………………………………….
   - o Not applicable (I did not experience any of the above complaints)

The following questions are about <u>current</u> complaints.

7. Do you <u>currently</u> experience urinary complaints, which you think are due to cauda equina syndrome? (for example: urinary incontinence, inability to urinate, reduced or absent sensation of passing urine, urinary catheter)
   - o Yes, namely:……………………………………………….
   - o No

8. Do you <u>currently</u> experience bowel complaints, which you think are due to cauda equina syndrome? (for example: incontinence, constipation, reduced or absent sensation of passing stool)
   - o Yes, namely:……………………………………………….
   - o No

9. Do you <u>currently</u> experience sexual problems, which you think are due to cauda equina syndrome? (for example: unable to get an erection, difficulty or inability to reach orgasm, decreased sensation during sex)
   - o Yes, namely:………………………………………………..
   - o No

10. Did you receive any information from the neurosurgeon - either before or after surgery - about the recovery of urinary complaints, bowel complaints and sexual problems? (multiple answers possible)
    - o Information about recovery of urinary complaints;
    - o Information about recovery of bowel complaints;
    - o Information about recovery of sexual problems;
    - o I did not receive any of the above information.

11. Would you have appreciated more information from the neurosurgeon about the recovery of urinary complaints, bowel complaints and sexual problems due to cauda equina syndrome?
    - o Yes
    - o No

12. Do you appreciate to hear about the results of this study?
    - o Yes, by email. My email address: ………..………….
    - o Yes, by mail (hard copy)
    - o No.

If any of the indicated answers are unclear to us, we would like to telephone you for additional information. In case you do not have any objections, we kindly ask you to report your telephone number here so we can reach if necessary.
………………………………………………………………………………………………………
